# Abdominal perfusion pressure in critically ill cirrhotic patients: a prospective observational study

**DOI:** 10.1038/s41598-023-34367-6

**Published:** 2023-05-26

**Authors:** Rui Antunes Pereira, André F. Esteves, Filipe S. Cardoso, Rui Perdigoto, Paulo Marcelino, Faouzi Saliba

**Affiliations:** 1 Unidade de Cuidados Intensivos Polivalente 7 (UCIP7), Hospital de Curry Cabral, Centro Hospitalar Universitário Lisboa Central, Lisbon, Portugal; 2 Serviço de Medicina, Hospital Garcia de Orta, Almada, Portugal; 3 Unidade de Transplante, Hospital de Curry Cabral, Centro Hospitalar Universitário Lisboa Central, Nova Medical School, Lisbon, Portugal; 4 Unidade de Cuidados Intensivos Polivalente 4 (UCIP4), Hospital de Santa Marta, Centro Hospitalar Universitário Lisboa Central, Lisbon, Portugal; 5 AP-HP Hôpital Paul Brousse, Hepato-Biliary center, Université Paris Saclay, INSERM unit Nº 1193, Villejuif, France

**Keywords:** Gastroenterology, Medical research, Risk factors

## Abstract

In critical patients, abdominal perfusion pressure (APP) has been shown to correlate with outcome. However, data from cirrhotic patients is scarce. We aimed to characterize APP in critically ill cirrhotic patients, analyze the prevalence and risk factors of abdominal hypoperfusion (AhP) and outcomes. A prospective cohort study in a general ICU specialized in liver disease at a tertiary hospital center recruited consecutive cirrhotic patients between October 2016 and December 2021. The study included 101 patients, with a mean age of 57.2 (± 10.4) years and a female gender proportion of 23.5%. The most frequent etiology of cirrhosis was alcohol (51.0%), and the precipitant event was infection (37.3%). ACLF grade (1–3) distribution was 8.9%, 26.7% and 52.5%, respectively. A total of 1274 measurements presented a mean APP of 63 (± 15) mmHg. Baseline AhP prevalence was 47%, independently associated with paracentesis (aOR 4.81, CI 95% 1.46–15.8, p = 0.01) and ACLF grade (aOR 2.41, CI 95% 1.20–4.85, p = 0.01). Similarly, AhP during the first week (64%) had baseline ACLF grade (aOR 2.09, CI 95% 1.29–3.39, p = 0.003) as a risk factor. Independent risk factors for 28-day mortality were bilirubin (aOR 1.10, CI 95% 1.04–1.16, p < 0.001) and SAPS II score (aOR 1.07, CI 95% 1.03–1.11, p = 0.001). There was a high prevalence of AhP in critical cirrhotic patients. Abdominal hypoperfusion was independently associated with higher ACLF grade and baseline paracentesis. Risk factors for 28-day mortality included clinical severity and total bilirubin. The prevention and treatment of AhP in the high-risk cirrhotic patient is prudential.

## Introduction

Cirrhosis increases intrahepatic resistance and leads to impairment of hepatosplanchnic blood flow. These changes result in chronic portal vein hypertension, further aggravated, in advanced stages of the disease, by compensatory splanchnic vasodilation, relative hypotension and the development of ascites.

The pathophysiologic aspects of intra-abdominal hypertension (IAH), in the decompensated cirrhotic patient with ascites, have been previously studied, as well as the safety and immediate beneficial effects of therapeutic large volume paracentesis (LVP) on hemodynamic status and regarding renal, respiratory and hepatic functions^1–4^.

In critically ill patients, abdominal perfusion pressure (APP), resulting from the difference between mean arterial pressure (MAP) and intra-abdominal pressure (IAP), correlates with improved survival^10^. Furthermore, in the decompensated cirrhotic patient, APP correlates with the clearance of indocyanine green, and may be predictive of organ dysfunction and outcome^5,7^. Only a few studies have reported various clinical cut-off values for APP, ranging from 50 to 72 mmHg, and potential resuscitation endpoints have been proposed^6–10^. However, the clinical importance of APP, prevalence, risk factors and outcomes for abdominal hypoperfusion (AhP) require specific research in the area of the critically ill cirrhotic patient.

The objectives of this study were to characterize APP in a population of critically ill cirrhotic patients, to analyze the prevalence and risk factors of AhP, clinical outcomes, including mortality rates at 28 and 90 days, intensive care unit (ICU) and hospital length-of-stay (LOS).

## Methods

### Design, settings, participants and definitions

This was a single center prospective cohort study of cirrhotic patients admitted to the ICU.

The study was set in a general ICU, with 21 beds, specialized in liver disease, at Hospital de Curry Cabral, Centro Hospitalar Universitário Lisboa Central, Portugal, a tertiary hospital center with a liver transplant program.

Patients were recruited between October 2016 and December 2021 and followed-up to hospital discharge or to the last known date of patient record at the center.

Data was collected at admission and throughout ICU stay, and included demographic and clinical variables, for the calculation of general and liver specific severity scores, as well as liver cirrhosis etiology, precipitating event of acute illness, arterial blood lactate concentration and vital organ support with vasopressors, mechanical ventilation and renal replacement therapy (RRT) during ICU stay.

The study protocol was approved by the Ethics Committee at Centro Hospitalar Universitário Lisboa Central (CES nº397/2017), and waived the need for individual informed consent for this observational study. All study procedures followed the principles of the Declaration of Helsinki^11^.

All cirrhotic patients admitted in the ICU with a bladder catheter in situ were consecutively screened for eligibility to avoid selection bias and to maximize the number of cases in the study. Patient selection was performed using the following inclusion criteria: (1) age ≥ 18 years, (2) first ICU admission during the index hospital stay, and (3) medical type of admission (no surgery in the 4 weeks preceding the index ICU admission). The exclusion criteria were: (1) any type of surgical ICU admission, (2) contra-indication for intravesical IAP measurements, (3) absence of recorded APP values, (4) patients with ICU stay duration inferior to 24 h and (5) patients with previous LT.

Patient data was retrieved on site or from medical records and collected in an anonymous and protected database.

Cirrhosis was defined as bridging fibrosis on previous liver biopsy or a composite of clinical signs and findings provided by laboratory tests, endoscopy, and radiologic imaging^12^.

The definition of IAH and abdominal compartment syndrome (ACS), IAP measurement methodology and clinical management of these patients followed the published and updated guidelines by the World Society of Abdominal Compartment Syndrome (WSACS)^8,13,14^. Accordingly, IAH was classified into grade I-IV (respectively 12–15, 16–20, 21–25 and > 25 mmHg). Abdominal hypoperfusion (AhP) was defined by an APP < 60 mmHg and ACS was defined as IAP > 20 mmHg in this population of critically ill patients.

For this study, “Paracentesis” refers to both diagnostic and LVP, unless otherwise stated. Large-volume paracentesis was defined for a volume ≥ 500 mL of drained ascites. Post-paracentesis circulatory disfunction (PPCD) was actively prevented with 20% albumin (8 g/L of drained ascites) infusion, according to clinical guidelines, and standard-of-care fluid therapy to ensure euvolemic state.

Intra-abdominal pressure monitoring was performed via trans-bladder measurement technique with a maximum of 25 mL of saline solution and zero-pressure reference point was set at the phlebostatic axis in the midaxillary line^14^.

Measures were performed every 6–8 h, and mean APP (APP = MAP − IAP) was calculated on each day for each patient. Values presented for IAP, APP and MAP in this study correspond to daily mean value unless otherwise stated. The expressions "ICU admission" and "baseline" are interchangeable, and refer to the period corresponding to calendar day zero (0) and day one (1) of ICU stay, to assure completeness of 24-h ICU stay data. Whenever a patients underwent emergent liver transplant during the ICU stay, IAP measure and APP calculation were halted due to the change in the type (surgical) of patient. These patients were included in the overall mortality analysis at 28-days.

Outcome measurements included survival data at 28 and 90 days, and length-of-stay in the ICU and hospital.

### Statistical analysis

Chi-square test was used to compare the frequency of categorical variables for independent groups. Shapiro–Wilk test was used to assess for normal distribution of continuous variables. T-test was then used to compare the mean between two normally distributed groups and the median test to compare the median of non-normal continuous variables. Multivariate analysis was performed using backward stepwise logistic regression, and included variables based on clinical importance and with p value ≤ 0.10 in univariate analysis, after assessment of statistical assumptions, namely, independence of observations, absence of influential outliers, linearity in the logit for continuous variables and collinearity, using Kendall Tau coefficient to identify and exclude strongly correlated predicting variables (above + 0.35 or less than − 0.35)^15^. The area under the Receiver operator curve (aROC) was used to determine the ability of a continuous variable to discriminate between a dichotomous outcome and the Younden's J statistic (Younden index) was used to identify the optimal cut-off value. Statistical significance was considered for two-sided p value ≤ 0.05. Statistical software IBM SPSS Statistics for Windows, version 23.0. Armonk, NY was use for analysis.

## Results

### Overall

**Figure 1:**
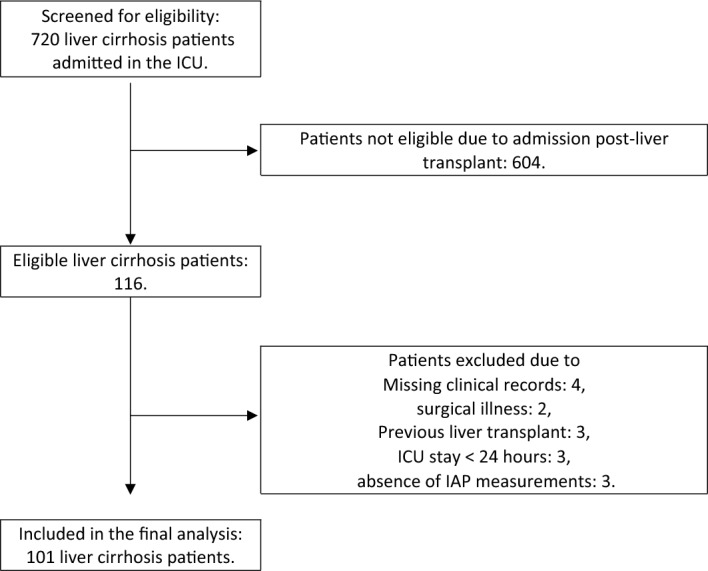
Study patient flowchart.

This study included 101 cirrhosis patients. A patient flowchart is depicted in Fig. 1.

Patients presented a mean age of 57.2 (± 10.4) years and female gender represented 23.5% of the cohort. Liver disease etiology included alcohol alone (51.0%), alcohol plus hepatitis C virus (HCV) (13.7%), NASH (5.9%), HCV alone and non-C viral hepatitis (both 4.9%). There was a history of past liver disease decompensation in 62.7% of patients, and hepatic related comorbidities included any type of hepatic neoplasm (20.6%), portal vein thrombosis (18.6%) and ascites (88.2%). The most frequent precipitant events leading to index ICU admission were infection (37.3%), bleeding (23.5%), hepatic encephalopathy (7.8%) and acute kidney injury (6.9%). The need for vasopressor therapy was present in 75% of patients at baseline, namely, noradrenaline (30%), or terlipressine (18%), or both in combination in 27% of cases. Clinical severity at ICU admission presented a mean SAPS II of 49 (± 15.0), median MELD-Na of 31 [23, 37], mean CLIF-C of 53 (± 11) and acute-on-chronic liver failure (ACLF) grades (I–III) of 8.9%, 26.7% and 52.5%, respectively. The observed mortality rates were 48.1% in-ICU and 64.4% in-hospital, and 56.4% and 67.6%, at 28 and 90 days, respectively. Median (days) ICU stay was 8 [4, 12] and hospital stay 23 [14, 42]. During the hospital stay liver transplant was performed in 18 (17.8%) cases after index ICU admission (Table 1).

**Table 1:** Baseline clinical characteristics in liver cirrhosis patients intensive care and 28-day vital outcome comparison.

Baseline variables	Overall	Non-survivor at day 28	Survivor at day 28	p
N	101	57	44	
Age (years)	57.1 (10.4)	57.0 (11.3)	57.4 (9.4)	0.8
Male gender, n (%)	77 (76.2)	45 (78.9)	32 (72.7)	0.6
Liver disease etiology, n (%)				0.14
Alcohol	52 (51.5)	29 (50.9)	23 (52.3)	
Alcohol + HCV	14 (13.9)	5 (8.8)	9 (20.5)	
Precipitant, n (%)				0.6
AKI^a^	7 (6.9)	4 (7.0)	3 (6.8)	
Bleeding	24 (23.8)	13 (22.8)	11 (25.0)	
Encephalopathy	8 (7.9)	6 (10.5)	2 (4.5)	
Infection	38 (37.6)	23 (40.4)	15 (34.1)	
CRP (mg/L)	51 [18, 93]	50 [18, 93]	56 [18, 84]	0.9
WBC count (10^3/mL)	11.9 [6.4, 18.3]	13.5 [8.4, 19.1]	8.5 [5.4, 15.7]	0.02
Hematocrit (%)	23.9 (5.7)	23.8 (5.9)	24.0 (5.6)	0.9
INR	2.2 [1.7, 3.1]	2.40 [1.78, 3.60]	1.85 [1.60, 2.42]	0.004
Platelets (10^3/mL)	67 [42, 121]	67 [46, 146]	62 [38, 94.25]	0.3
Urea (mg/dL)	90 [55, 131]	97 [62, 146]	70.50 [45.75, 105]	0.03
Creatinine (mg/dL)	1.8 [0.9, 3.0]	2.1 [1.3, 3.1]	1.3 [0.8, 2.6]	0.04
Urine output (mL/24h)	1090 [498, 1823]	1005 [418, 1695]	1133 [805, 1924]	0.5
Bilirubin (total, mg/dL)	6.0 [2.3, 17.6]	11.1 [4.9, 24.5]	3.5 [1.9, 6.4]	< 0.001
Ammonia (ug/dL)	240 [159, 314]	243 [177, 230]	189 [137, 306]	0.2
West-Haven score	1 [0, 3]	3 [1, 3]	1 [0, 2]	0.001
PaO2/FiO2 ratio	257 [170, 356]	257 [166, 356]	249 [178, 354]	0.9
Arterial blood pH (minimum)	7.38 [7.30, 7.43]	7.35 [7.28, 7.42]	7.41 [7.35, 7.45]	0.03
Lactate (mmol/L)	2.6 [1.5, 4.3]	2.9 [1.9, 5.3]	2.2 [1.4, 3.5]	0.01
Fluid balance (mL)	1618 [433, 3623]	1699 [− 410, 3744]	1563 [715, 2908]	0.9
Ascites, n (%) (n = 98)	87 (86.1)	4885.7)	38 (90.5)	0.7
Paracentesis, n (%)	38 (37.6)	23 (40.4)	15 (34.1)	0.7
Paracentesis volume (mL)^b^ (n = 21)	3000 [1800, 4500]	4000 [1825, 5575]	2300 [1640, 2900]	0.2
SAPS II score	49 (15)	53 (16)	43 (11)	< 0.001
MELD Na score	31 [23, 37]	34 [28, 40]	26 [17, 32]	< 0.001
ACLF grade	3 [2, 3]	3 [2, 3]	2 [1, 3]	< 0.001
AKI^a^, n (%)	66 (65.3)	44 (77.2)	22 (50.0)	0.008
RRT, n (%)	24 (23.8)	16 (28.1)	8 (18.2)	0.4
IMV, n (%)	56 (55.4)	36 (63.2)	20 (45.5)	0.12
Vasopressors, n (%)	72 (71.3)	41 (71.9)	31 (70.5)	1
IAP (mmHg)	12 [8, 15]	13 [9, 15]	11 [8, 14]	0.2
MAP (mmHg)	72 [66, 81]	72 [63, 77]	75 [68, 90]	0.2
APP (mmHg)	63 (15)	60 (14)	67 (15)	0.050
Mortality at day 28, n (%)	57 (56.4)			
ICU LOS (days)	8 [4, 12]	7 [4, 10]	9 [6, 16]	0.06
Hosp stay (days)	24 [14, 42]	19 [8, 25]	39 [24, 65]	< 0.001

*IAP* intra-abdominal pressure, *APP* abdominal perfusion pressure, *BMI* body mass index, *HCV* Hepatitis C virus, *ACLF* acute-on-chronic liver failure, *INR* international normalization ratio, *CRP* C-reactive protein, *PaFiO2* arterial oxygen partial pressure to fractional inspired oxygen ratio, *IMV* invasive mechanical ventilation, *AKI* acute kidney injury, *RRT* renal replacement therapy, *SAPS II* simplified acute physiology score II, *CLIF-C* Chronic Liver Failure Consortium, *MELD-Na* Model For End-Stage Liver Disease—sodium, *MAP* mean arterial pressure, *ICU* intensive care unit, *LOS* length-of-stay, *WBC* white blood cell.

^a^Diagnosis of AKI as indicated in clinical records.

^b^Paracentesis volume (mL) includes only large-volume paracentesis (≥ 500 mL), and excludes diagnostic paracentesis.

Normally distributed continuous variables are presented as mean (SD) and non-normal continuous variables as median [IQR]. IAP and MAP values are drawn from pooled APP data components.

A total of 1274 APP measurements were recorded throughout the ICU stay, approximately, corresponding to a mean of 13 per patient. Measured pressures (mmHg) presented a median IAP of 12.4 [9.6, 13.9], a mean MAP of 78.2 (± 11.0) and a median APP of 65.9 [58.6, 72.0]. The distribution of IAH grades (I–IV) during the ICU stay and associated mortality rates are depicted in Supplementary Fig. 1.

### Paracentesis

Paracentesis was performed in 38% of patients at ICU admission, and LVP (≥ 500 mL) (n = 21) presented a median volume of 3000 mL [1800, 4500]. Additionally, the frequency of paracentesis performed prior to ICU admission was 37%, after D1 of ICU stay was 51%. In 27% of cases there was no record of paracentesis during the entire hospital stay and this was justified due to absent/minimal ascites (n = 16) or waived based on a confirmed clinical diagnosis (n = 9, including pneumonia, hydrothorax and ruptured esophageal varices).

**Figure 2:**
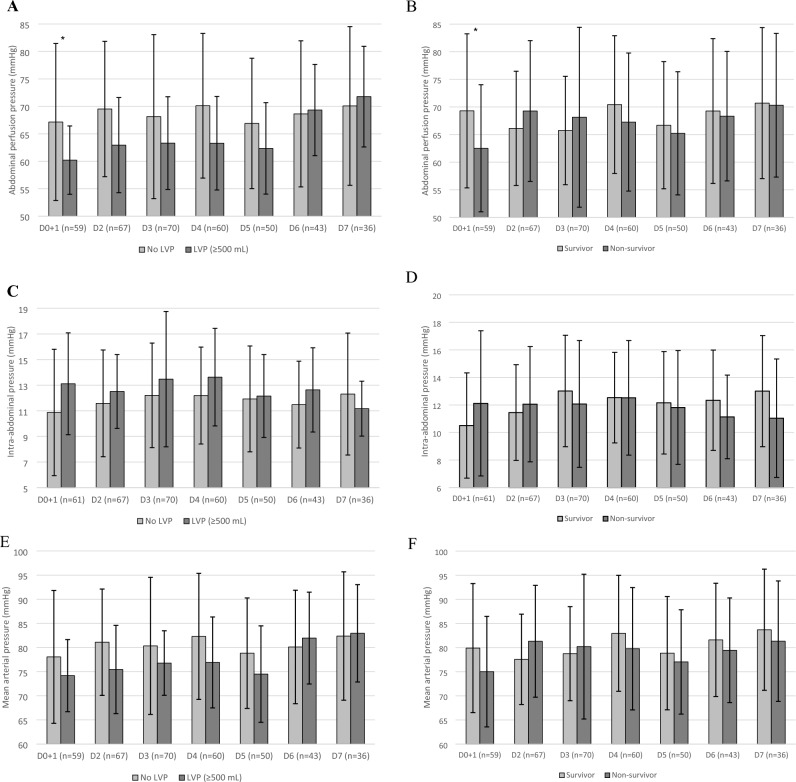
Critical pressures during the first 7 days of intensive care. Abdominal perfusion pressure (**A**, **B**), intra-abdominal pressure (**C**, **D**), mean arterial pressure (**E**, **F**). *Statistically significant (p ≤ 0.05) intraday difference between groups. Patients submitted to large-volume paracentesis (≥ 500 mL) at baseline (n = 21) were compared to all other study patients (n = 79). Mortality was assessed at day 28; survivors: n = 43, non-survivors: n = 57. Error bars: ± 1 standard deviation. *LVP* large-volume paracentesis, *D* Day.

At baseline, APP was significantly lower in patients submitted to LVP (60 ± 6.2 vs. 67 ± 14, p = 0.01) when compared to the rest of the patients and did not significantly differ throughout the rest of the study period days (Fig. 2). The variation in APP from baseline to D2 was not significantly different between patients with/without baseline LVP at ICU admission (p = 0.9).

Large-volume paracentesis (> 500 mL, n = 21) was not associated with baseline AhP (p = 0.3) nor with 28-day mortality (p = 0.4) in multivariate analysis.

### Abdominal hypoperfusion

**Figure 3:**
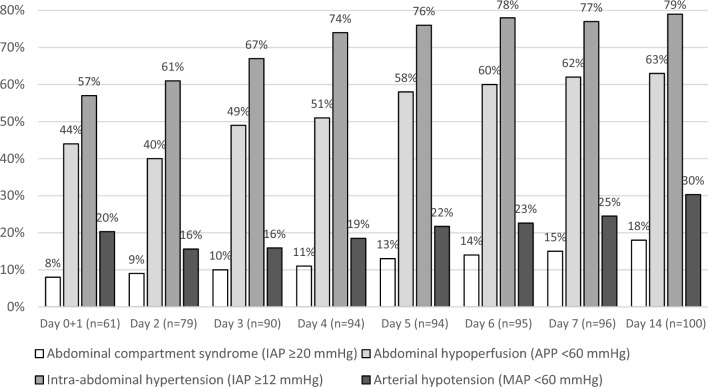
Cumulative prevalence of critical pressures in the cirrhotic patient in intensive care. The frequencies shown take into account the daily mean values. Abbreviations: *D* day, *IAP* intra-abdominal hypertension, *APP* abdominal perfusion pressure, *MAP* mean arterial pressure.

Baseline AhP had a prevalence of 47%, as illustrated in Fig. 3, and was associated with higher serum urea concentration and clinical severity SAPS II score, lower arterial blood pH and the presence of paracentesis in univariate analysis (Table 2). Multivariate analysis (n = 59) revealed independent association of any type of paracentesis (both diagnostic and LVP) (aOR 4.81, CI 95% 1.46–15.8, p = 0.01) and ACLF grade (aOR 2.41, CI 95% 1.20–4.85, p = 0.01) with AhP at baseline (Table 3).

**Table 2:** Comparison of abdominal perfusion pressure groups in liver cirrhosis patients in intensive care.

Baseline variables	At ICU admission			During ICU stay		
	APP < 60 mmHg	APP ≥ 60 mmHg	p	APP < 60 mmHg	APP ≥ 60 mmHg	p
N (%)	28 (47.5)	31 (52.5)		67 (67.7)	32 (32.3)	
Age (years)	58 [52, 63]	58 [52, 63]	0.9	58 (11)	55 (9)	0.13
Male gender, n (%)	22 (78.6)	25 (80.6)	1.0	53 (79.1)	23 (71.9)	0.6
Liver disease etiology, n (%)			0.8			0.3
Alcohol	15 (53.6)	15 (48.4)		39 (58.2)	12 (37.5)	
Alcohol + HCV	4 (14.3)	4 (12.9)		9 (13.4)	5 (15.6)	
Precipitant, n (%)			0.9			0.2
AKI^a^	2 (7.1)	4 (12.9)		3 (4.5)	4 (12.5)	
Bleeding	5 (17.9)	5 (16.1)		13 (19.4)	10 (31.2)	
Encephalopathy	3 (10.7)	2 (6.5)		6 (9.0)	2 (6.2)	
Infection	12 (42.9)	11 (35.5)		26 (38.8)	12 (37.5)	
CRP (mg/L)	50 [23, 94]	27. [13, 66]	0.2	56 [18, 95]	47 [18, 88]	0.6
WBC count (10^3/mL)	13.2 [6.5, 20.0]	10.8 [6.5, 16.7]	0.3	12 [6.8, 18.4]	11.3 [6.4, 17.9]	0.7
Hematocrit (%)	24.8 (5.9)	23.4 (6.8)	0.4	24.40 (5.71)	23.0 (5.8)	0.2
INR	2.4 [1.7, 2.8]	2.2 [1.7, 2.9]	0.7	2.3 [1.7, 3.0]	1.9 [1.6, 3.5]	0.4
Platelets (10^3/mL)	70 [45, 126]	54 [43, 93]	0.3	69 [41, 99]	56 [41, 139]	0.9
Urea (mg/dL)	115 [78, 178]	72 [44, 110]	0.01	93 [62, 145]	66 [41, 98]	0.02
Creatinine (mg/dL)	2.3 [1.5, 2.9]	1.3 [0.8, 2.9]	0.10	2.1 [1.0, 3.2]	1.2 [0.8, 2.2]	0.01
Urine output (mL/24h)	975 [393, 2053]	1268 [898, 1933]	0.4	970 [409, 1503]	1330 [930, 1923]	0.03
Bilirubin (total, mg/dL)	6.2 [2.4, 12.0]	6.7 [3.4, 20.2]	0.5	5.70 [2.26, 13.96]	6.58 [2.52, 19.04]	0.8
Ammonia (ug/dL)	238 [170, 286]	255 [169, 308]	0.7	228 [159, 333]	255 [163, 294]	0.7
West-Haven score	2 [0, 3]	1 [0, 3]	0.6	1 [0, 3]	2 [0, 3]	0.3
PaO2/FiO2 ratio	253 [157, 354]	286 [166, 357]	0.6	257 [163, 355]	257 [197, 359]	0.7
Arterial blood pH (minimum)	7.34 [7.24, 7.40]	7.40 [7.32, 7.44]	0.03	7.36 [7.29, 7.42]	7.40 [7.36, 7.47]	0.02
Lactate (mmol/L)	2.9 [1.9, 9.1]	2.5 [1.3, 3.7]	0.09	2.7 [1.7, 4.3]	2.3 [1.5, 4.1]	0.2
Fluid balance (mL)	1565 [115, 4405]	819 [− 396, 2194]	0.3	1791 [273, 4104]	1426 [563, 2624]	0.4
Ascites, n (%)	27 (100)	27 (87.1)	0.2	54 (90.0%)	28 (84.8)	0.7
Paracentesis, n (%)	18 (64.3)	10 (32.3)	0.03	29 (43.2)	9 (28.1)	0.11
Paracentesis volume (mL)^b^ (n = 21)	3500 [1650, 5613]	3110[2055, 4375]	0.9	3500 [1750, 5538]	2220[1850, 2600]	0.4
SAPS II score	56 (17)	43 (14)	0.004	50 (16)	46 (13)	0.3
MELD Na score	31 [26, 37]	29 [20, 38]	0.5	31 [25, 37]	28 [19, 40]	0.3
ACLF grade	3 [2, 3]	2 [1, 3]	0.06	3 [2, 3]	2 [1, 3]	0.03
AKI^a^, n (%)	22 (78.6)	17 (54.8)	0.10	47 (70.1)	17 (53.1)	0.2
RRT, n (%)	6 (21.4)	8 (25.8)	0.9	18 (26.9)	6 (18.8)	0.5
IMV, n (%)	18 (64.3)	12 (38.7)	0.09	37 (55.2)	18 (56.2)	1.0
Vasopressors, n (%)	24 (85.7)	19 (61.3)	0.07	52 (77.6)	18 (56.2)	0.05
IAP (mmHg)	13 [11, 15]	11 [7, 14]	0.04	13 [9, 15]	9 [7, 14]	0.2
MAP (mmHg)	65 [60, 71]	81 [72, 92]	< 0.001	71 [62, 79]	81 [74, 97]	0.002
APP (mmHg)	51 (7)	73 (11)	< 0.001	59 (13)	76 (12)	< 0.001
Mortality at day 28, n (%)	21 (75.0)	14 (45.2)	0.04	39 (58.2)	17 (53.1)	0.8
ICU LOS (days)	7 [2, 12]	8 [4, 11]	0.2	9 [5, 14]	7 [4, 9]	0.03

*IAP* intra-abdominal pressure, *APP* abdominal perfusion pressure, *BMI* body mass index, *HCV* Hepatitis C virus, *ACLF* acute-on-chronic liver failure, *INR* international normalization ratio, *CRP* C-reactive protein, *PaFiO2* arterial oxygen partial pressure to fractional inspired oxygen ratio, *IMV* invasive mechanical ventilation, *AKI* acute kidney injury, *RRT* renal replacement therapy, *SAPS II* simplified acute physiology score II, *CLIF-C* Chronic Liver Failure Consortium, *MELD-Na* Model For End-Stage Liver Disease – sodium, *MAP* mean arterial pressure, *ICU* intensive care unit, *LOS* length-of-stay, *WBC* white blood cell.

^a^Diagnosis of AKI as indicated in clinical records.

^b^Paracentesis volume (mL) includes only large-volume paracentesis (≥ 500 mL), and excludes diagnostic paracentesis.

Normally distributed continuous variables are presented as mean (SD) and non-normal continuous variables as median [IQR]. IAP and MAP values are drawn from pooled APP data components.

**Table 3:** Multivariate analysis for independent associations with the presence of abdominal hypoperfusion in intensive care.

	Baseline variables	p	Odds ratio	95% CI	
				Lower	Upper
AhP at baseline (n = 59)^a^	Paracentesis	0.01	4.81	1.46	15.8
	ACLF grade	0.01	2.41	1.20	4.85
AhP up to day 7 (n = 92)^b^	Paracentesis	0.07	2.51	0.92	6.86
	ACLF grade	0.003	2.09	1.29	3.39

Abdominal hypoperfusion corresponds to a daily mean abdominal perfusion pressure < 60 mmHg.

*AhP* abdominal hypoperfusion, *C.I.* confidence interval, *ACLF* acute-on-chronic liver failure.

^a^Multivariate analysis included: urea, pH, lactate, invasive mechanical ventilation, ACLF grade and paracentesis.

^b^Multivariate analysis included: creatinine, urine output, pH, paracentesis and ACLF grade.

During the entire ICU stay, AhP presented a cumulative prevalence of 63% (Fig. 3) and was associated with higher creatinine and urea concentrations, urine output, ACLF grade and lower pH at baseline (Table 2). Multivariate analysis (n = 92), with the addition of paracentesis of any type at admission due to clinical relevance, revealed that higher ACLF grade (aOR 2.09, CI 95% 1.29–3.39, p = 0.003) was significantly associated with AhP during the first week of ICU stay, differently from paracentesis (aOR 2.51, CI 95% 0.92–6.86, p = 0.07) (Table 3).

### Mortality

Baseline APP (mmHg) was lower in non-survivors at 28-days when compared to survivors (60 ± 14 vs. 67 ± 15, p = 0.050) (Table 1), and those with AhP had a higher 28-day mortality rate (75.0% vs. 45.2%, p = 0.04) (Table 2). Additionally, mortality was also associated with WBC, bilirubin, urea, SAPS II score, lactate, West-Haven score, paracentesis, INR, creatinine, pH and a clinical diagnosis of AKI at admission. (Table 1) In multivariate analysis (n = 96) we observed that bilirubin (aOR 1.10, CI 95% 1.04–1.16, p < 0.001) and SAPS II score (aOR 1.07, CI 95% 1.03–1.11, p = 0.001) were independently associated with 28-day mortality. Similar results were observed for 90-day mortality. When we included baseline AhP, due to clinical importance, in the multivariate analysis (n = 55) West-Haven hepatic encephalopathy score was the only risk factor for 28-day mortality (Table 4).

**Table 4:** Multivariate analysis for 28-day mortality risk factors in liver cirrhosis patients in intensive care.

	Baseline variables	p	Odds ratio	95% CI	
				Lower	Upper
Mortality at 28 days (n = 96)	Lactate	0.4	1.07	0.92	1.24
	C-reactive protein	0.07	1.01	1.00	1.02
	SAPS II	0.048	1.04	1.00	1.09
	Bilirubin	0.001	1.13	1.05	1.21
Variables included: white blood cell count, bilirubin, urea, SAPS II score, lactate, West-Haven score, paracentesis at admission					
Mortality at 28 days (n = 55)	Baseline AhP	0.09	0.31	0.08	1.20
	SAPS II	0.08	1.04	1.00	1.09
	West-Haven score	0.02	1.88	1.11	3.21
Variables included: white blood cell count, bilirubin, urea, SAPS II score, lactate, West-Haven score, paracentesis and AhP at baseline					

Paracentesis corresponds to both diagnostic and large-volume paracentesis.

*C.I.* confidence interval; *SAPS* simplified acute physiologic score; *AhP* abdominal hypoperfusion

Baseline APP presented a poor ability to discriminate between survivors and non-survivors at 28 days (aROC of 0.64, CI 95% 0.50–0.79, p = 0.07) (Supplementary Fig. 2) with an optimal cut-off value of ≥ 59 mmHg (Younden index 0.28).

Finally, patients presenting AhP had a longer ICU LOS (days) (9 [5, 14] vs. 7 [4, 9], p = 0.03) (Table 2), compared to those without AhP.

## Discussion

### Overall

This is the largest study to address the impact of APP on clinical outcomes in critically ill cirrhotic patients^5,6,16^. The typical patient in our cohort was a 57-year-old male with alcoholic liver disease and ascites, admitted in intensive care with ACLF grade 3.

The main findings of this study were: (1) a high prevalence of AhP, in approximately half of the population at baseline and in two thirds of patients during the first week of ICU stay, (2) AhP was independently associated with higher clinical severity, (3) these patients were five times more likely to be submitted to paracentesis at baseline, and (4) 28-day mortality risk factors included higher clinical severity, total bilirubin and hepatic encephalopathy at ICU admission.

Chronically increased IAP is present in the physiological state of pregnancy^17–20^ and in pathological states such as morbid obesity^21,22^, decompensated heart failure^23–25^ and liver cirrhosis^26,27^.

Specific clinical thresholds for IAP and APP in the cirrhotic patient are yet to be defined, particularly, since advanced cirrhosis with portal hypertension leads to multiple compensatory mechanisms. These include a hyperdynamic state (due to splanchnic and systemic arterial vasodilation resulting in reduced effective blood volume) with compensatory vasoconstriction and reduced organ perfusion, cardiomyopathy, microvascular and endothelial dysfunction. Furthermore, systemic inflammation, mitochondrial dysfunction, oxidative stress and metabolic changes can lead to tissue injury and extrahepatic organ failure^28^. These mechanisms potentially modify pathophysiologic responses to acute critical illness as seen in other types of patients and illnesses (i.e., acute pancreatitis, major burns and abdominal surgery).

In our cohort of critically ill liver cirrhosis patients the overall mean APP baseline value was low when compared to other populations of intensive care patients^29,30^. In a mixed population of 100 intensive care patients, where 42% of patients had IAH, the overall mean APP value was 74 (± 17) mmHg^17^. In another study, 50 patients with severe acute pancreatitis had a mean APP of 80 (± 5) mmHg^18^. Comparatively, our cohort presented lower APP with differences of − 11 and − 17 mmHg, respectively.

Nearly two thirds of our patients had AhP during the ICU stay. Two studies in critically ill cirrhotic patients reported a high prevalence of AhP^5,6^. In the first study, Al-Dorzi et al. analyzed 61 septic shock patients, reporting a prevalence of AhP of 70% at ICU admission. Interestingly, an APP of 55 mmHg was identified as the best cut-off value to discriminate survivors from non-survivors, and AhP was not significantly associated with any of the studied outcomes in multivariate analysis. This study concluded that IAH was associated with increased ICU morbidity and mortality, although no independent risk factors for IAH were found^5^. In the second study, Mayr et al. reported a prevalence of AhP between 25 and 50% of cases (inferred from a median APP value of 63 [57, 70] mmHg, n = 22), and was able to quantify hepatosplanchnic blood flow impairment due to IAH^6^. Our study confirms a high prevalence of AhP among critically ill cirrhotic patients^6^.

### Abdominal hypoperfusion

Acute-on-chronic liver failure severity score was predictive of AhP at baseline and during the first week of ICU stay. This reflected the severity of our typical ACLF grade 3 patient with shock and multiorgan failure, frequently treated with noradrenaline and terlipressine perfusions combined. Whereas, for less severe upper gastrointestinal bleeding and hepato-renal syndrome, terlipressine was the preferred vasoactive agent. Three quarters of our cohort received vasopressor therapy, nonetheless, arterial hypotension persisted in one fifth of patients at baseline. In our view, higher clinical severity with the presence of shock combined with increased IAP was the main reason for the association between higher ACLF grade and AhP.

Patients with AhP were five times more likely to be submitted to paracentesis at baseline, although, we did not observe an improvement in the ensuing daily APP variation when compared to the rest of the patients without paracentesis at baseline, nor did we find and association between LVP and AhP. We speculate these results signaled an increased clinical awareness for diagnostic screening of spontaneous bacterial peritonitis and the treatment and prevention of IAH in these high-risk patients. Particularly, since PPCD preventive measures were standard-of-care, a higher fluid balance at ICU admission was observed in the AhP. Although we did not find an association between LVP and AhP, an increase in APP (with a decrease of IAP and central venous pressure, without change in circulating volume), as well as improved hepatosplanchnic blood flow, has been described after LVP. This was corroborated by ultrasound hepatic artery resistance index, hepatic vein maximum flow velocity, and indocyanine green plasma disappearance rate (positively correlated to APP and inversely correlated to IAP), considered a dynamic surrogate marker of hepatic perfusion and hepatocellular function^6,7,31,32^. Importantly, patients not submitted to paracentesis were clinically justified, inasmuch as the use of paracentesis has been suggested as a key inpatient quality measure in cirrhosis^33,34^.

### Mortality

Baseline APP was lower in non-survivors than in survivors at 28 days, although it presented an inadequate discriminatory ability. Abdominal hypoperfusion was not a risk factor for mortality in our cohort, probably due to the small sample size and the multifactorial nature of critical illness. Unambiguously, mortality was associated with baseline clinical severity and total bilirubin, reflecting the dual character of the “acute” critical illness and the “chronic” liver disease in this population of cirrhotic patients. Additionally, West-Haven hepatic encephalopathy score was the only independent risk factor for 28-day mortality in the subset of patients with available baseline APP data. This highlights the vital importance of acute neurologic disfunction in critically ill cirrhotic patients, as previously reported^16,34,35^.

Furthermore, patients with AhP had longer ICU LOS, indicating greater patient comorbidity and higher associated healthcare costs.

We support the rationale for considering APP a critical vital sign and to use it to further assist the clinician in titrating volume repletion, vasopressor use and optimizing IAP, thus preventing deleterious effects of persistent critical pressures^9,10,14,36,37^. The treatment and prevention of AhP in high-risk cirrhotic patients is prudential.

### Limitations

Limitations in this study include a relatively small sample size, due to slow recruitment aggravated by the COVID-19 pandemic onset, and missing baseline APP data due to work load and delayed patient enrolment into the study protocol. Additionally, the lack of longitudinal data on organ dysfunction and the impact of therapies aimed at optimizing APP precluded further results and outcome analysis. The impact of AhP on specific organ failures should be specifically addressed in the future.

Strengths of this study include the fact that it is the largest prospective study addressing APP in consecutive critically ill cirrhotic patients, this way minimizing selection bias, provides data on the impact of baseline paracentesis in the critically ill cirrhotic patient, and opens the field for further research^38^. Future studies on AhP, IAH and ACS should focus on the first week of ICU admission^38,39^.

## Conclusion

This study confirms a high prevalence of AhP in critically ill cirrhotic patients. Abdominal hypoperfusion was independently associated with higher ACLF grade and paracentesis performed at ICU admission.

Mortality at 28 days was higher among patients with AhP and independent risk factors were higher clinical severity, total bilirubin and hepatic encephalopathy.

Abdominal perfusion pressure can be considered a critical vital sign and prevention and treatment of AhP in the high-risk cirrhotic patient is prudential.

## Supplementary Information

Supplementary Figure 1.

Supplementary Figure 2.

## Data Availability

The datasets used and analyzed during the current study are available from the corresponding author on reasonable request.
