# Supplementary material for: Abdominal perfusion pressure in critically ill cirrhotic patients: a prospective observational study
Source: Sci Rep. 2023 May 26;13:8550. doi: 10.1038/s41598-023-34367-6 (PMC10214359; doi:10.1038/s41598-023-34367-6)
Supplement: Supplementary file 1 — Supplementary Figure 1. [file 41598_2023_34367_MOESM1_ESM.docx]

Supplementary figure 1. Intra-Abdominal Hypertension and mortality rates in the cirrhotic patient in intensive care.

Figure 3 caption. Distribution of IAH grades during the entire ICU stay (n=101). IAH grades take into account the highest daily mean value of intra-abdominal pressure throughout the ICU stay. Abbreviations: IAH, intra-abdominal hypertension.
