# Supplementary material for: Abdominal perfusion pressure in critically ill cirrhotic patients: a prospective observational study
Source: Sci Rep. 2023 May 26;13:8550. doi: 10.1038/s41598-023-34367-6 (PMC10214359; doi:10.1038/s41598-023-34367-6)
Supplement: Supplementary file 2 — Supplementary Figure 2. [file 41598_2023_34367_MOESM2_ESM.docx]

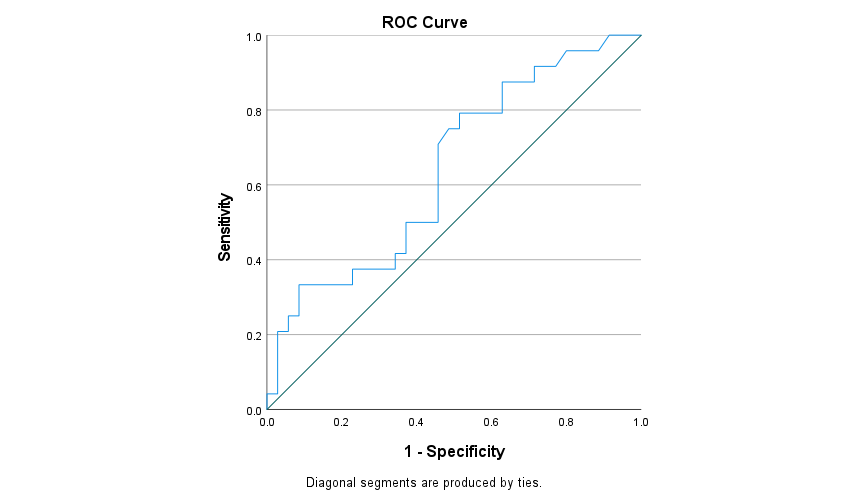


| **Area Under the Curve** | | | | |
| --- | --- | --- | --- | --- |
| Test Result Variable(s): D01_APP_mean | | | | |
| Area | Std. Error^a^ | Asymptotic Sig.^b^ | Asymptotic 95% Confidence Interval | |
|  |  |  | Lower Bound | Upper Bound |
| .642 | .073 | .065 | .499 | .785 |
| The test result variable(s): D01_APP_mean has at least one tie between the positive actual state group and the negative actual state group. Statistics may be biased. | | | | |
| a. Under the nonparametric assumption | | | | |
| b. Null hypothesis: true area = 0.5 | | | | |

Supplementary figure 2. Receiver operator curve for the ability of baseline abdominal perfusion pressure to discriminate 28-day vital outcome.
